# Simultaneous Assessment of Electroencephalography Microstates and Resting State Intrinsic Networks in Alzheimer's Disease and Healthy Aging

**DOI:** 10.3389/fneur.2021.637542

**Published:** 2021-06-17

**Authors:** Stefan J. Teipel, Katharina Brüggen, Anna Gesine Marie Temp, Kristina Jakobi, Marc-André Weber, Christoph Berger

**Affiliations:** ^1^German Center for Neurodegenerative Diseases (DZNE), Rostock, Germany; ^2^Department of Psychosomatic Medicine, Rostock University Medical Center, Rostock, Germany; ^3^Institute of Diagnostic and Interventional Radiology, Pediatric Radiology and Neuroradiology, Rostock University Medical Center, Rostock, Germany; ^4^Department of Psychiatry, Neurology, Psychosomatics, and Psychotherapy in Childhood and Adolescence, Rostock University Medical Center, Rostock, Germany

**Keywords:** EEG, fMRI, Alzheimer's disease, brain function, resting state activity

## Abstract

Electroencephalography (EEG) microstate topologies may serve as building blocks of functional brain activity in humans. Here, we studied the spatial and temporal correspondences between simultaneously acquired EEG microstate topologies and resting state functional MRI (rs-fMRI) intrinsic networks in 14 patients with Alzheimer's disease (AD) and 14 healthy age and sex matched controls. We found an anteriorisation of EEG microstates' topologies in AD patients compared with controls; this corresponded with reduced spatial expression of default mode and increased expression of frontal lobe networks in rs-fMRI. In a hierarchical cluster analysis the time courses of the EEG microstates were associated with the time courses of spatially corresponding rs-fMRI networks. We found prevalent negative correlations of time courses between anterior microstate topologies and posterior rs-fMRI components as well as between posterior microstate topology and anterior rs-fMRI components. These negative correlations were significantly more expressed in controls than in AD patients. In conclusion, our data support the notion that the time courses of EEG microstates underlie the temporal expression of rs-fMRI networks. Furthermore, our findings indicate that the anterior-to-posterior connectivity of microstates and rs-fMRI components may be reduced in AD, indicative of a break-down of long-reaching intrahemispheric connections.

## Introduction

Preclinical findings of synaptic dysfunction from *ex vivo* studies in rodent brain slices (1–4) and *in vivo* studies in animal models of cerebral amyloidosis (5) suggest that Alzheimer's disease (AD) pathology affects neuronal function and connectivity. The blood-oxygenation level dependent (BOLD) signal in functional MRI (fMRI) can be used to assess neuronal functional connectivity *in vivo*. Consistent with the preclinical findings on synaptic dysfunction, fMRI studies found alterations of intrinsic resting state networks in AD patients compared with controls. These intrinsic networks reflect spontaneous fluctuations of the BOLD signal during rest conditions (6) and correspond to networks of functional activation during specific task performances (7). Several studies have reported reduced spatial expression of the so-called default mode network (DMN) and other resting state networks in people with AD dementia or amnestic mild cognitive impairment (MCI), an at-risk stage of AD (8–11).

Resting state EEG is another functional imaging modality that has widely been used to characterize functional brain changes in AD (12). EEG studies showed reductions of high frequency and increase of low frequency power as well as impaired interhemispheric coherence in AD patients compared with controls (13, 14). In 1987, Lehmann et al. identified so called microstates from multichannel EEG recordings: spatially consistent patterns of scalp potential topographies that remain stable for about 100 milliseconds before changing into another topography (15). More recently, the number of underlying topographies was found to be finite and classes of these microstates were found to be reproducible across sessions and subjects (16). Spatial clustering identified four classes of microstates that accounted for up to 80% of total topographic variance in resting state EEG; for a contemporary review see (17). Lehmann has suggested “EEG-defined functional microstates as basic building blocks of mental processes” (18).

Microstates' duration, field power and transition probabilities can be influenced by brain diseases, such as schizophrenia (19), and frontal lobe dementia (20). In AD patients, the occurrence and duration of microstate toplogies deviates from healthy controls (21–26). Most of these studies showed a stronger expression of anterior vs. posterior microstate topologies in AD patients compared with controls.

The simultaneous analysis of microstates is particularly interesting in conjunction with rs-fMRI, because the microstates' topography may represent a high temporal resolution correlate of the intrinsic resting state networks obtained by rs-fMRI (27, 28). Thus, the simultaneous analysis of EEG microstates with rs-fMRI may reveal dynamic underpinnings of altered spatial and temporal expression of intrinsic resting state networks in AD.

In the current study, we used simultaneously acquired EEG and rs-fMRI data to investigate whether the time courses of EEG microstate topologies correlated with time courses of spatially corresponding rs-fMRI networks, and if these correlations differed between AD patients and controls. More specifically, we expected that spatial pattern of microstates would correspond with spatial pattern of rs-fMRI. We anticipated that more frontal and more posterior activity in EEG would be associated with more frontal and more posterior activity in rs-fMRI, respectively, and that these associations would be less pronounced in AD cases than in controls due to the expected degradation of functional connectivity in AD.

These analyses from simultaneously acquired EEG and fMRI data allow direct investigation if pattern of microstates topologies underlie the temporal expression of resting state fMRI networks, and provide insight into AD-related differences in coupling between microstates and rs-fMRI networks. Due to the lack of previous evidence, our analyses will serve to generate hypotheses on specific associations of EEG and resting state fMRI in AD which can be tested in subsequent studies.

## Methods

### Participants

We recruited 18 patients with mild AD via the memory clinic at the Rostock University Medical Center, and 17 cognitively healthy older controls via the Rostock University Medical Center database. Healthy controls were required to score within one standard deviation on all subscales of the Consortium to Establish a Registry for Alzheimer's Disease (CERAD) battery (29). Patients were clinically diagnosed with probable AD dementia according to the NINCDS-ADRDA and NIA-AA criteria (30). However, one patient aborted the scan session, three patients were excluded due to radiological abnormalities, and three women in the control group had to be randomized out to match the groups for sex. The final participants were 14 individuals with a clinical diagnosis of AD dementia and 14 cognitively healthy older controls, matched for age, sex and education. All subjects underwent general medical, neurological and psychiatric assessment. Neuropsychological assessment was conducted using the CERAD battery. Laboratory analyses and APOE genotype sequencing were carried out. Participants exhibited no non-AD-related neurological or radiological abnormalities (e.g., normal pressure hydrocephalus or extensive microinfarcts, vascular dementia), and no psychiatric diseases. Eight AD patients took antidementive medication with cholinesterase inhibitors, one with Memantine, five took no antidementive medication. The study was approved by the local ethics committee. All participants gave written informed consent, and all procedures were carried out in accordance with the Helsinki declaration in its present form.

### Data Acquisition

Electroencephalography and fMRI data were recorded simultaneously during 7.5 min of resting state (eyes-closed). For the EEG recording, MRI-comaptible Brain Amp and the software Brain Vision Recorder 1 (both from Brain Products, Gilching, Germany) were used. EEG was recorded at 32 electrodes that were positioned according to the international 10–20-system (31). The reference electrode was located between Fz and Cz, the ground electrode at AFz. Impedances of the electrodes of interest (O1, O2, and Oz) were kept below 8 kΩ, except for one AD patient (18 kΩ). An additional ECG channel was attached to detect cardio-ballistic artifacts. EEG data were sampled at 5 kHz. The EEG amplifier sampling interval was phase-synchronized to the fMRI main frequency via the Syncbox (Brain Products, Gilching, Germany) in order to preclude EEG-fMRI-sampling-jitter artifacts. The EEG amplifier and powerpack were placed at the head end of the scanner tube and weighted with sand bags to prevent hardware motion.

Functional magnetic resonance imaging images were acquired using a 3-Tesla Magnetom Verio scanner (Siemens Healthineers, Erlangen, Germany) with a T2-weighted echo-planar imaging sequence (TR: 2.6 s, TE: 30 ms, FOV: 224 mm, thickness: 3.5 mm, number of slices: 47, number of volumes: 180). Participants were instructed to stay awake and keep their eyes closed throughout the entire fMRI sequence. The EEG signal was visually controlled for signs of sleep (offline). The anatomical images were recorded using a T1-weighted MPRAGE sequence (TR: 2.5 s, TE: 4.37 ms, FOV 256 mm, thickness: 1 mm, number of slices: 192). Foam wedges were used to stabilize the head.

### Data Preprocessing

#### MRI Data

Functional magnetic resonance imaging data preprocessing was performed using SPM8 and the VBM8 toolbox (Version 4143) in Matlab 7 (Mathworks, Natick). The first six volumes were removed to eliminate T1-saturation effects. Slices were referenced to the middle slice, temporally-speaking. After realigning the functional images, the anatomical images were co-registered to the realigned mean functional image. The structural T1-weighted MPRAGE images were segmented into gray matter, white matter and cerebrospinal fluid compartments and warped to standard MNI space, using the default MNI standard template and the Diffeomorphic Anatomical Registration Through Exponentiated Lie Algebra (DARTEL) method (32), implemented in VBM8. The resulting deformation fields were used to warp the functional images to standard space.

Rs-fMRI networks were calculated using the FSL melodic toolbox (Version 5.0.9, FMRIB, Oxford, UK, http://www.fmrib.ox.ac.uk/fsl/) with a preset number of 30 independent component analysis (ICA) maps. We applied spatial smoothing with a 5 mm isotropic full-width-at-half-maximum (FWHM) Gaussian kernel and a high-pass filter with a cut-off period of 128 s. The resulting maps were visually evaluated to identify the resting-state networks most closely resembling the four topologies of the microstates. We further derived the subject-level rsFC z-maps using FSL's dual regression, which generated subject-specific versions of the spatial maps and associated time series. This was realized by a decomposition of each subject's 4D dataset using the group-spatial-maps to give a set of time courses, and followed by decomposition of those time courses and the same 4D dataset to get one subject-specific spatial map per functional network (33, 34).

#### EEG Data

EEG data were preprocessed using Brain Vision Analyzer software (Version 2.0, Brain Products, Gilching, Germany). Firstly, MRI gradient-artifacts in EEG data were corrected using the average artifact subtraction method (35) with a sliding average of 21 baseline-corrected intervals from all channels as the sliding template to remove from the EEG data. Corrected EEG data were then down-sampled to 250 Hz. ECG pulse artifacts were removed by constructing an average ECG artifact template of 21 ECG pulses and subtracting it from the EEG data. Data were bandpass-filtered between 0.5 and 70 Hz including an additional notch filter at 50 Hz. Artifacts caused by eye movement, temporal electrode noise and residual pulse artifacts were removed using ICA. In case the electrode noise could not be eliminated by removing two independent components, the disturbed channel was removed and interpolated by topographical triangulation. After ICA, the data were visually inspected for residual artifacts. No sleep patterns (i.e., K-complexes or sleep spindles) were present. EEG data from the AD group showed more artifacts such as eye movement and muscle activation, especially during the second half of the scan time, possibly constituting a sign of growing unrest. Two AD subjects showed a shift in frequency from alpha to theta over time, indicative of declining vigilance. These vigilance-related artifacts were removed. Data were bandpass-filtered again between 1 and 30 Hz and were re-referenced to a common reference, obtained by averaging across all channels. EEG power in Delta (1–3.5 Hz), Theta (3.5–8.2 Hz), Alpha (8.2–14 Hz) and Beta (14–30 Hz) band was calculated using complex demodulation (36) and pooled across all channels. Complex demodulation includes firstly the so-called “demodulation,” i.e., the shifting of the frequency spectrum of the EEG time series toward the origin by the frequency of interest. Secondly, all signal parts above the frequency of interest were low pass-filtered. Let the time-dependent amplitude be A, and phase P of the periodic signal of interest at f_0_ and the rest Z(t). Then, a time series can be determined by X(t) = A(t) cos(2πf_0_t+P(t)) + Z(t). Signal shift then means multiplying by exp(–i2πf_0_t) resulting in Y(t) = ½ A(t) exp{iP(t)} + ½ A(t) exp(–i4πf_0_t + P(t)) + Z(t) exp(–i2πf_0_t). The low pass filter removes signal at or above f_0_ resulting in Y'(t) = ½ A'(t) exp(iP'(t)). From this, we extracted the power A'=2|Y'|. Complex demodulation has shown to be the most effective and flexible method for envelope extraction from real signals (37). The preprocessed multichannel EEG time courses as well as the pooled EEG power data were segmented into artifact-free intervals spanning 2 s and exported for further analysis.

## Microstate Analysis

Microstates of EEG data were calculated and their statistical properties were derived using the Microstate toolbox (38), a plugin for the EEGLAB toolbox (39). Briefly, microstates analysis includes firstly the identification of the topographical microstates prototypes at the peaks of the global field power (GFP, the spatial standard deviation between the electrodes), called “microstates segmentation.” Secondly, EEG data were labeled point-by-point with the class of the prototypes that was most similar. Similarity decision was based on the global map dissimilarity (40, 41) which measures the distance between topographies, and is invariant to the strength of the signals. Signals are calculated as the mean of the Euclidean distance between prototype and EEG sample, both normalized with GFP and referenced to average reference. After this back-fitting procedure, the microstate labels were temporally smoothed and the microstate statistics were calculated. The following settings were used: data selection (normalization: no, minimal peak distance: 10 milliseconds, number of peaks: 2,000, GFP threshold: 1 standard deviation, segmentation limited to healthy subjects), and microstate segmentation (normalization: no, algorithm: modified K-means, optimized iteration scheme). The modified K-means algorithm (42), used for topographical clustering, is invariant to map polarity and therefore will cluster two proportional but opposite maps together. The number of microstates for segmentation was selected based on the cross-validation criterion (CV) (42) which is related to the residual noise of the labeled EEG data. The clustering with the lowest CV represents the best solution. In our EEG data, the microstate clustering with four microstate classes had the lowest CV. Temporal smoothing of back-fitted labels was based on the small segment rejection method (38), with minimum microstate duration of 30 ms. This smoothing method changes the label of the EEG time points to the next most likely microstate class until the minimum microstate duration threshold is reached. Details of these clustering, validation and smoothing methods can be found in the Microstate toolbox guide (38).

The following microstate features were exported for further statistical analyses: the number of occurrences of each microstate class per second, the mean duration of each microstate in milliseconds, and the microstates' band power.

## Statistical Analysis

Between-group differences in age, education, and Mini Mental Status Examination (MMSE) scores were determined using Student's *t*-test. Effects on microstates' occurrences, duration and band power were determined using mixed measures ANOVA with the four microstate types as the within-subjects factor and diagnosis as between-subjects factor (AD vs. controls). In addition to *p*-values we estimate the effect sizes with ω^2^. Its interpretation resembles that of partial η^2^. However, with small sample sizes partial η^2^ tends to overestimate the variance explained; ω^2^ is less biased, i.e., it is always smaller than partial η^2^. Significant effects of diagnosis or diagnosis by microstate type were followed up using between-groups *t*-tests, using Cohen's d as effect size estimate. These effect sizes may be large (ω^2^ = 0.14, Cohen's *d* = 0.8), medium (ω^2^ = 0.06, Cohen's *d* = 0.5) or small (ω^2^ = 0.1, Cohen's *d* = 0.2). These analyses were conducted using IBM SPSS Statistics version 27.

Between-group differences in the spatial expression of rs-fMRI ICA components were determined using voxel-based partial least squares (PLS) analysis (43) using the PLS software (Rotman Research Institute, https://www.rotman-baycrest.on.ca/index.php?section=84) in Matlab R2013a. This approach operates on the covariance between brain voxels and allows the assessment of an integrated network of brain regions that co-varies with any external measure of choice (44), here the diagnosis. PLS is a multivariate analysis that operates across the entire brain and therefore requires no correction for multiple comparisons across multiple voxels. PLS analysis yields the latent variable (LV) which identifies a pattern of brain regions that conjointly covaries with diagnosis. Each voxel of the brain has a weight on each LV (“salience”). To reduce the number of models, we determined PLS effects of diagnosis only for the ICA maps that were visually rated to be spatially most similar to the corresponding microstate topology. Since we formulated an a priori assumption on the direction of the expected effect of diagnosis on each ICA component (more or less expressed in AD compared with controls, respectively) based on the direction of the effect of diagnosis on the corresponding microstate topology, we used a one-sided test, corresponding to a *p* < 0.1 threshold based on permutations tests with 500 permutations. We used bootstrap estimation (with 500 iterations) to determine the reliability of the saliences for the brain voxels determining each LV. The bootstrap ratios of salience follow a standard z-score distribution, where a ratio of > 1.96 corresponds to a *p*-value of < 0.05. In contrast to univariate analysis, the permutation tests and the saliences were determined in one single analytical step, rendering multiple comparison correction unnecessary.

For the analysis of the time course of rs-fMRI components and microstate's expression we used FSLnets (https://fsl.fmrib.ox.ac.uk/fsl/fslwiki/FSLNets), a collection of Matlab scripts with an interface to fsl graphical functions and fsl randomize. Using this toolbox, we produced a full correlation matrix between the rs-fMRI components that were identified to be spatially similar to the microstate topographies and these microstate's time courses. This matrix then was used for unsupervised clustering of time courses using an agglomerative hierarchical cluster tree with Ward's linkage through the Matlab command “linkage” (https://de.mathworks.com/help/stats/linkage.html#mw_08b425f7-fc8c-480a-b618-f768817e8e11).

Finally, we determined between-group differences in the correlation coefficients after Fisher's Z transformation using a linear model, with FDR correction via FSL randomize. We report FDR-corrected and uncorrected *p*-values for group differences in time course correlations, focusing on correlations between microstates and rs-fMRI components.

## Results

### Demographic Differences Between AD Patients and Controls

Group matching for sex, age and education was successful (all *p* > 0.2). As expected, AD patients had lower MMSE scores than controls (see Table 1 for details). One of the 14 AD patient could not complete the full rs-fMRI scan; consequently this case was included in the analysis of changes in microstates, but excluded from all analyses concerning rs-fMRI data.

**Table 1:** Demographic characteristics.

	**Sex distribution f/m**	**Mean age (SD) (years)^**a**^**	**Mean education (SD) (years)^**b**^**	**Mean MMSE score (SD)^**c**^**	**ApoE4 genotype no ε4/at least one ε4^**d**^**
AD patients	4/10	75.3 (5.7)	14.4 (2.7)	24.6 (3.1)	5/9
Controls	4/10	73.4 (3.1)	13.6 (2.8)	28.7 (0.8)	10/4

*f/m, female/male.*

*SD, standard deviation.*

a *Not significantly different between groups, T = −1.1, 26 df, p = 0.27.*

b *Not significantly different between groups, T = −0.8, 26 df, p = 0.42.*

c *Significantly different between groups, T = −4.8, 26 df, p < 0.001.*

d *Not significantly different between groups, Chi^2^ = 3.6, 1 df, p = 0.06*.

### Differences in Microstates' Occurrences and Duration Between AD Patients and Controls

**Figure 1:**
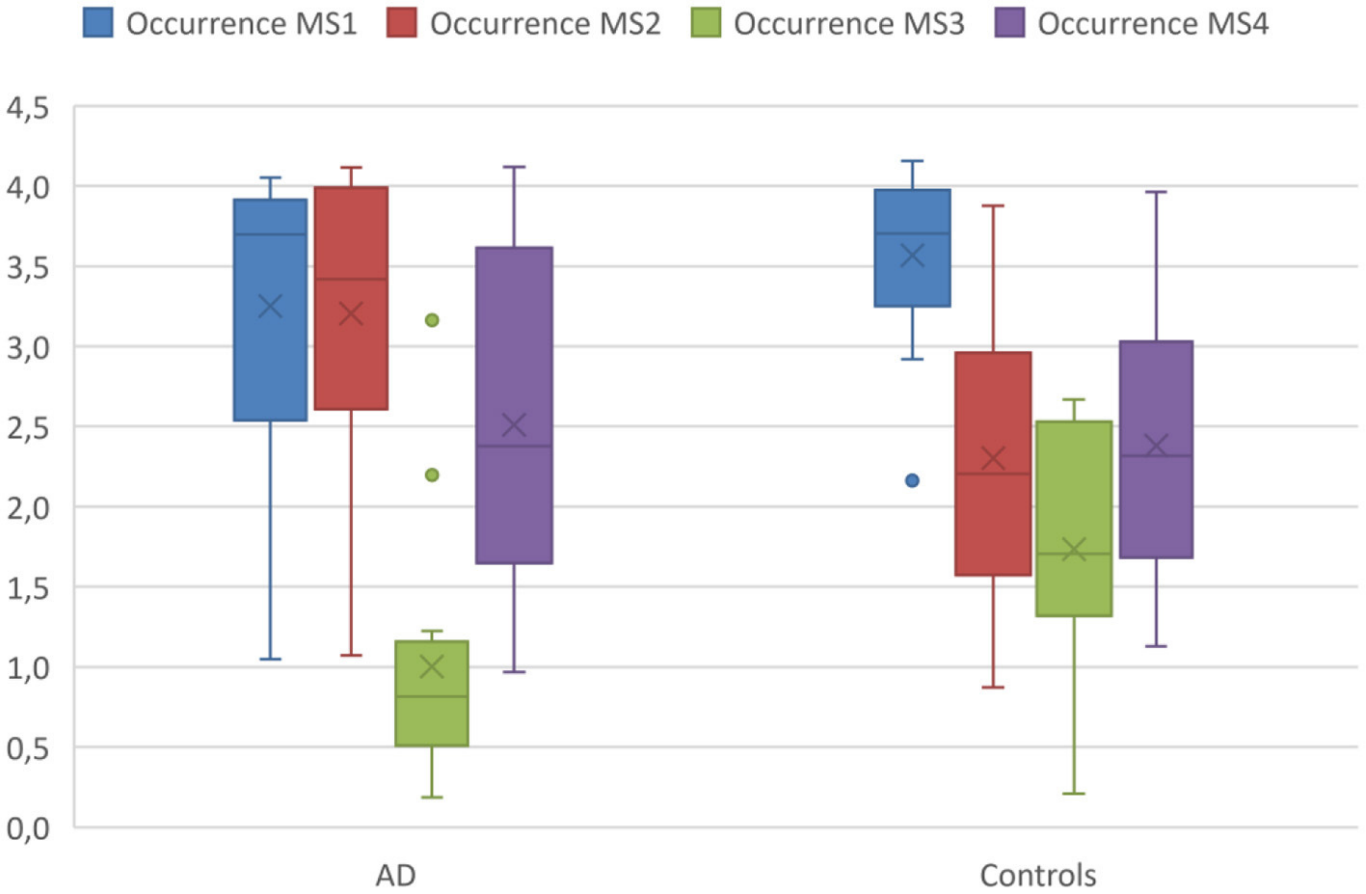
Occurrences of microstates in AD patients and controls. Boxplot of occurrences of microstates per second averaged across the entire acquisition period comparing the AD group with the controls. Boxplots show 1st quartile, median, and 3rd quartile as well as mean values (large cross).

Mixed ANOVA revealed a significant interaction of microstate class by diagnosis on occurrences [F_(3,78)_ = 3.84, *p* = 0.013, ω^2^ = 0.09], but not on duration [F_(3,78)_ = 2.02, *p* = 0.12, ω^2^ = 0.02] of microstates. *Post hoc* t-tests revealed the interaction to involve more occurrences of microstate 2 (T = 2.6, df = 26, *p* = 0.02, Cohen's d = −0.98) and fewer occurrences of microstate 3 (T = −2.5, df = 26, *p* = 0.02, Cohen's *d* = 0.95) in AD cases compared with controls (see Figure 1).

### Differences in Band Power of Microstates Between AD Patients and Controls

**Figure 2:**
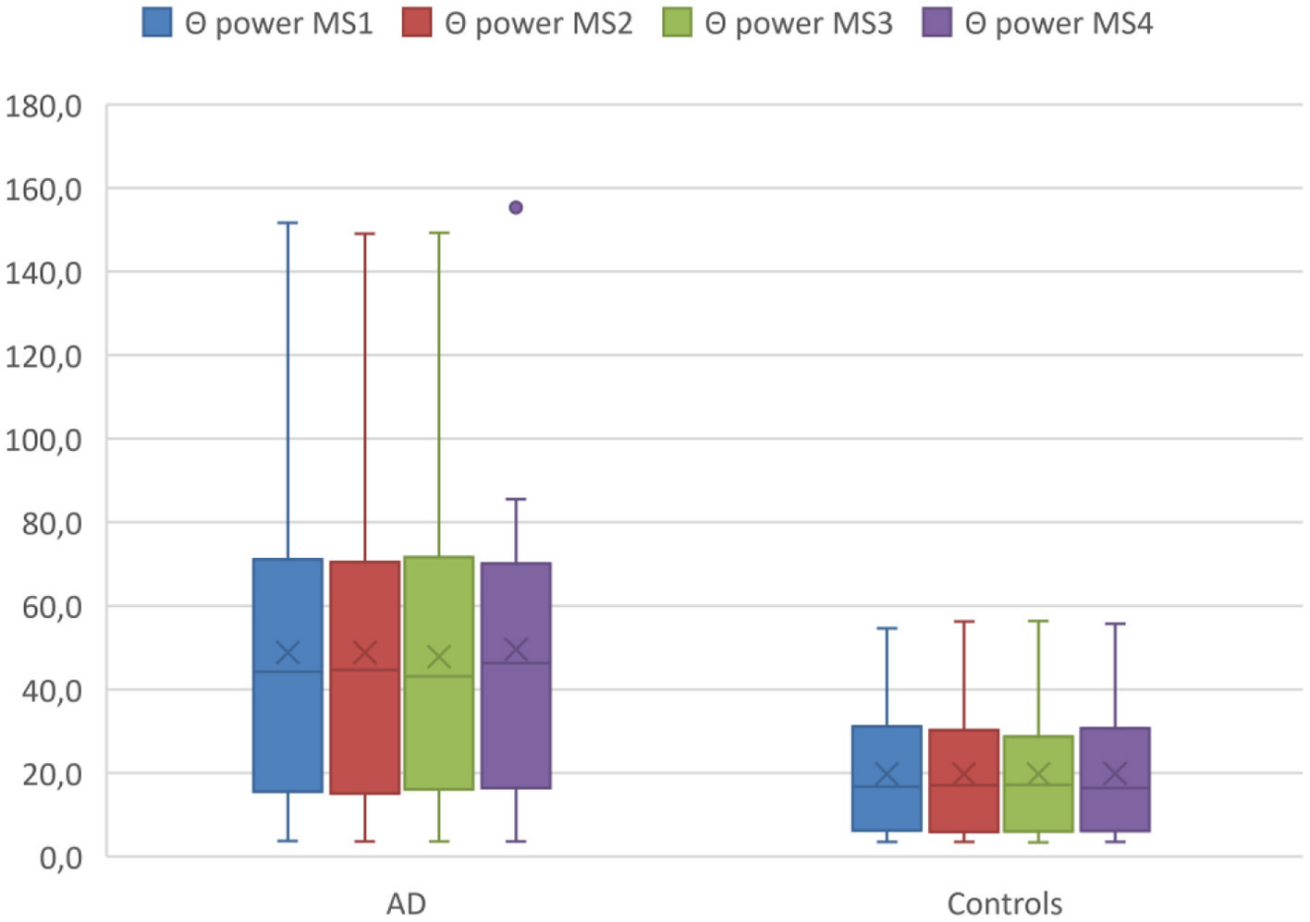
Theta (Θ) power within MS classes between diagnostic groups. Boxplot Theta (Θ) power of microstates averaged across the entire acquisition period comparing the AD group with the controls. Boxplots show 1st quartile, median, and 3rd quartile as well as mean values (large cross).

Using mixed ANOVA, we found significant effects of diagnosis across all microstate classes for delta power [F_(1,26)_ = 4.1, *p* = 0.05, ω^2^ = 0.10] and theta power [F_(1,26)_ = 6.7, *p* = 0.02, ω^2^ = 0.17], but no significant effects of group for alpha or beta power. *Post hoc* analyses revealed higher theta power for all microstate classes in AD patients compared with controls (T > 2.53, 26 df, *p* < 0.02 for all comparisons, Cohen's d < −0.96) (see Figure 2).

### Differences of Spatial Expression of Resting State fMRI Networks Between AD Patients and Controls

**Figure 3:**
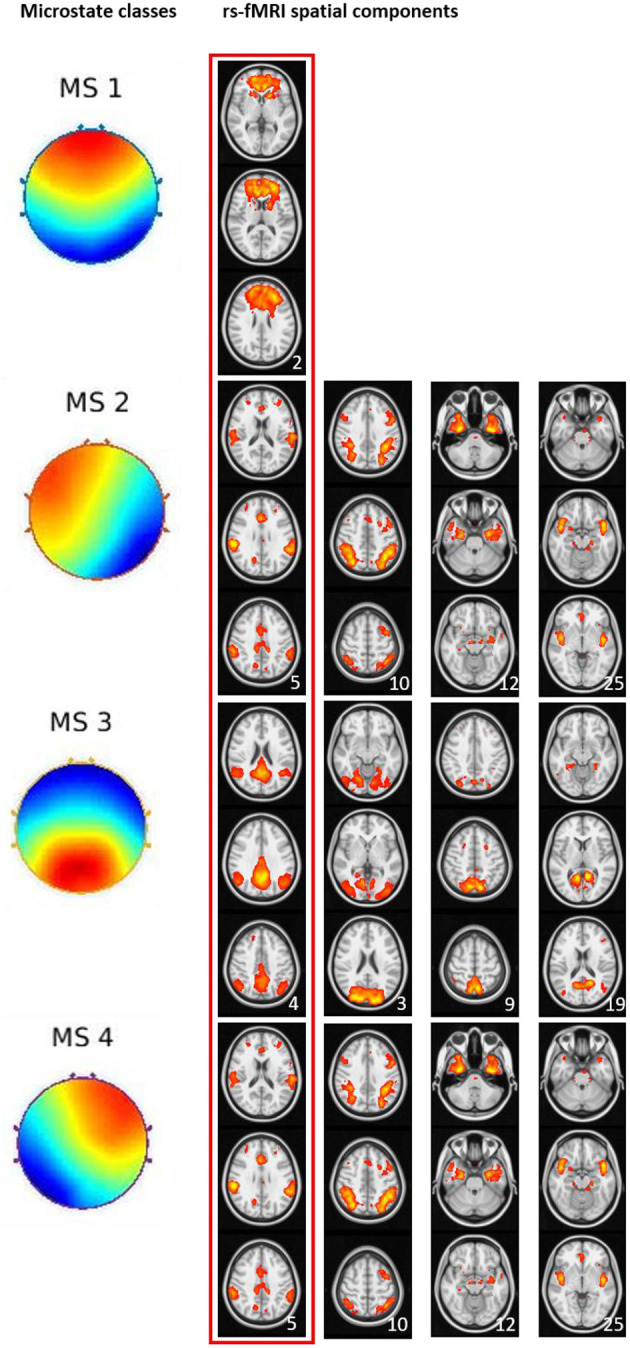
Visual match of microstate classes and spatial ICA components from rs-fMRI. Four microstate topologies and the spatially matching rs-fMRI components. Numbers in the lower right corner of each rs-fMRI component represent the position of this component among the 30 components from ICA analysis. The red box indicates the spatial rs-fMRI components that were visually rated to most closely resemble the microstate's topology.

**Figure 4:**
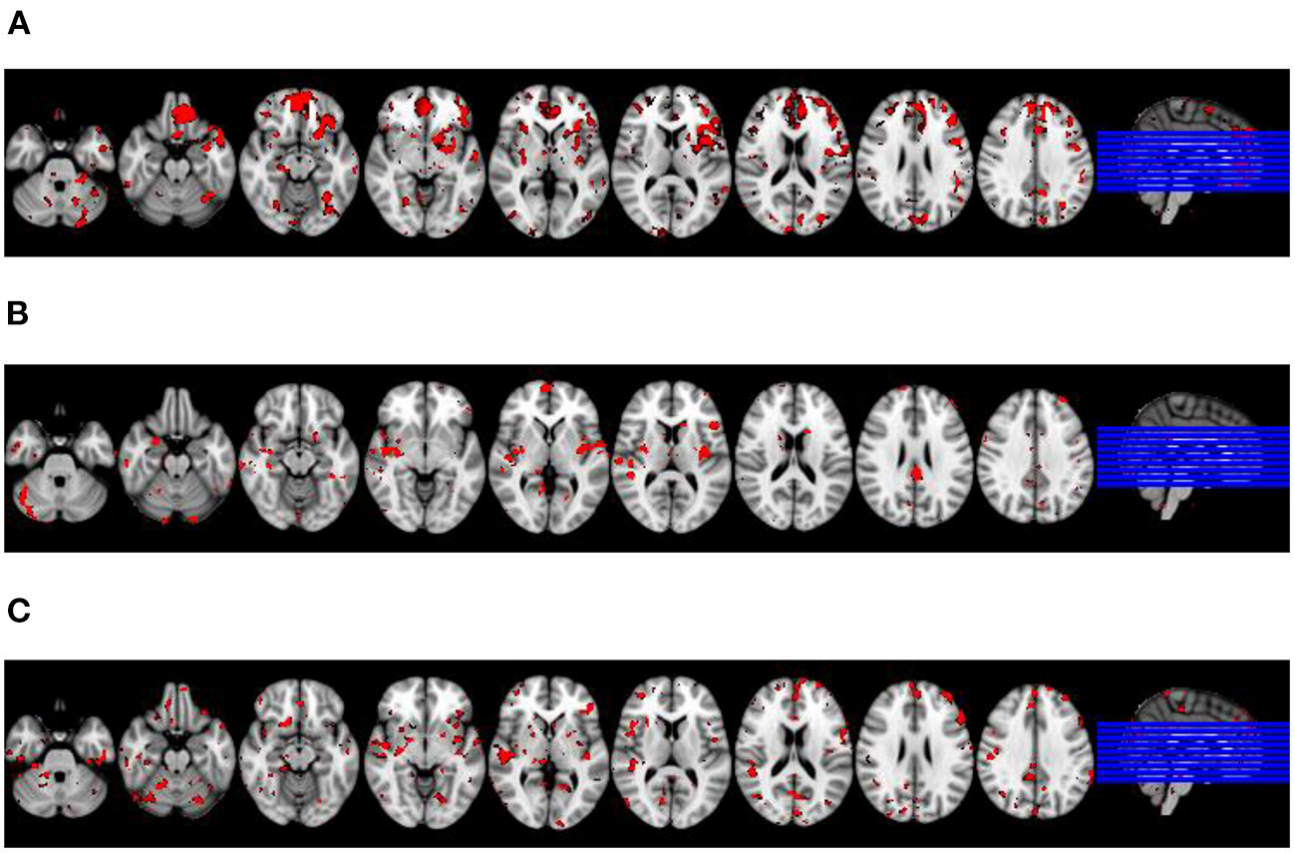
Differences in spatial expression of spatial ICA components between diagnostic groups. Latent variable representing brain regions where spatial expression of the respective rs-fMRI component was significantly associated with diagnosis projected on an MRI scan in MNI standard space. Axial sections go left to right from MNI coordinate *z* = −30 to z= 34; sections are 8 mm apart. Right of image is right of brain, view from superior. Red colored voxels represent a significant bootstrap ratio of *p* < 0.05, with an increased expression in **(A,C)**, and a decreased expression in **(B)**, respectively, in AD patients compared with controls. **(A)** Frontal lobe network. **(B)** Default lobe network. **(C)** Temporal lobe network.

In the first step, we visually identified spatial components for the melodic analysis of the controls' resting state fMRI data that resembled the spatial pattern of the microstate topologies (see Figure 3). To reduce the number of comparisons, we compared spatial expression of rs-fMRI components only for the components that were rated to be most similar to the corresponding microstate topologies. Based on the group differences in microstates 2 and 3 (Figure 1), we expected a higher spatial expression of the ICA component corresponding to microstate 2 and a lower spatial expression of the ICA component corresponding to microstate 3. The frontal lobe network corresponding to microstate 1 was spatially more expressed in AD cases than in controls, singular value 86.9, *p* < 0.10. The temporal lobe component, corresponding to microstates 2 and 4, was spatially more expressed in AD cases than in controls at a singular value of 83.5, *p* < 0.10. The default mode component, corresponding to microstate 3, was reduced in AD patients compared with controls, singular value 86.2, *p* < 0.10. The spatial effects are shown in Figure 4.

### Associations Between Time Courses of Microstates and Time Courses of BOLD Signal Within Resting State Networks

For these analyses, we used two different approaches to calculate time courses of the microstates: firstly, numbers of classifications of a microstate within a TR, and secondly, mean similarity of the EEG pattern to each of the microstates within a TR. We determined associations of microstates' time courses with the time courses of the nine rs-fMRI components identified by visual inspection to spatially resemble the microstates' topography. Based on the numbers of microstate classifications, hierarchical clustering in the combined groups revealed that time course of microstate 1 clustered with the frontal lobe rs-fMRI network. The time course of microstate 2 clustered with the time course of a bilateral medial and lateral temporal network, the time course of microstate 3 clustered with rs-fMRI components representing the default mode network and the visual network. Microstate 4 clustered with rs-fMRI components with a bilateral fronto-parietal and an occipital expression.

**Figure 5:**
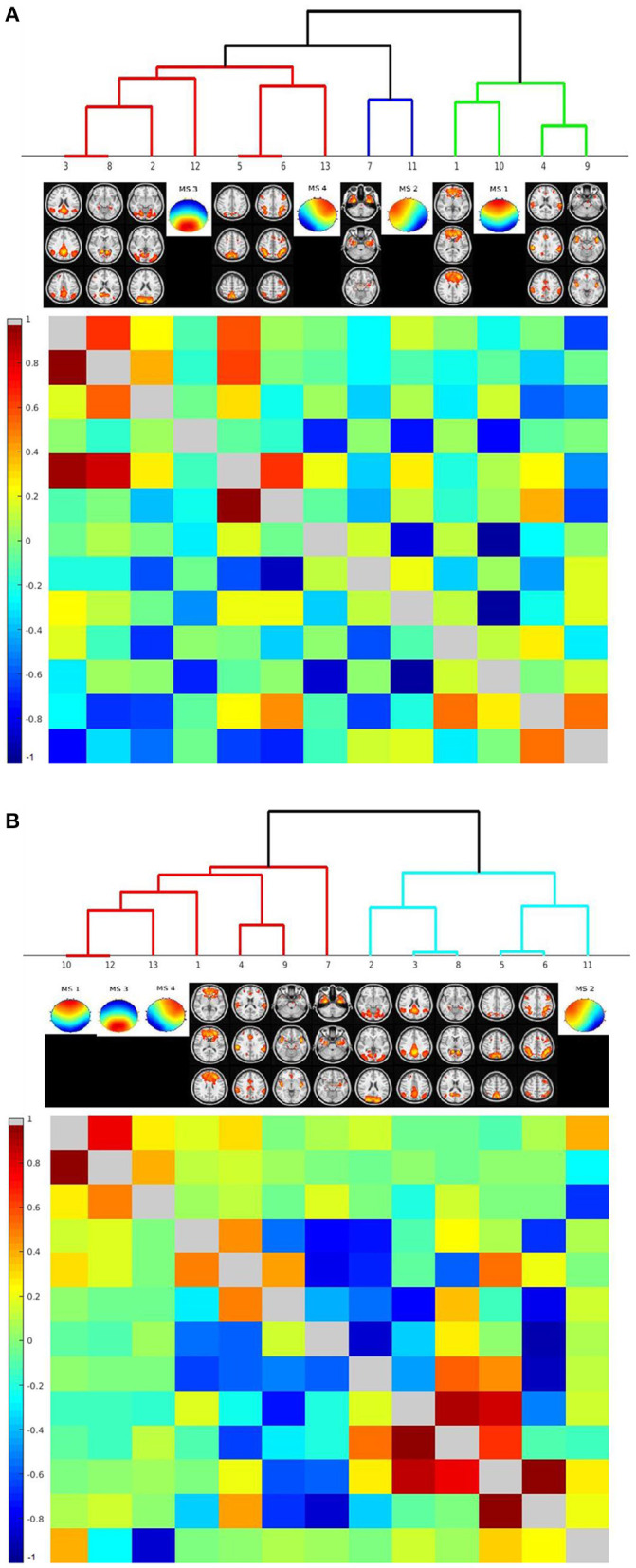
Hierarchical classification of rs-fMRI components' and microstates' time courses based on their cross-correlation across individuals. Cluster dendrograms for the time courses of rs-fMRI components and the microstates. The lower left triangular matrix indicates the cross-correlation of time courses, the upper right triangular matrix the partial correlations of time courses. Clustering was performed across all cases, including AD patients and controls. **(A)** Clustering based on number of assignments of microstates per TR. **(B)** Clustering based on similarity of EEG time courses with each microstate per TR.

Based on the similarity, the clustering was less well-defined. Microstate 2 clustered with the time course of the bilateral fronto-parietal network. Microstates 1, 3, and 4 clustered with each other and only at a higher level with frontal lobe network. All clustering results can be found in Figure 5.

When we repeated the clustering based on the data of the healthy controls only, we found similar results. The only exception was that the similarity time course of microstate 2 clustered with the bilateral medial and lateral temporal lobe network. Details can be found in Supplementary Figure 1.

### Differences of Correlations Between Microstate Components and rs-fMRI Networks Between AD Patients and Controls

**Figure 6:**
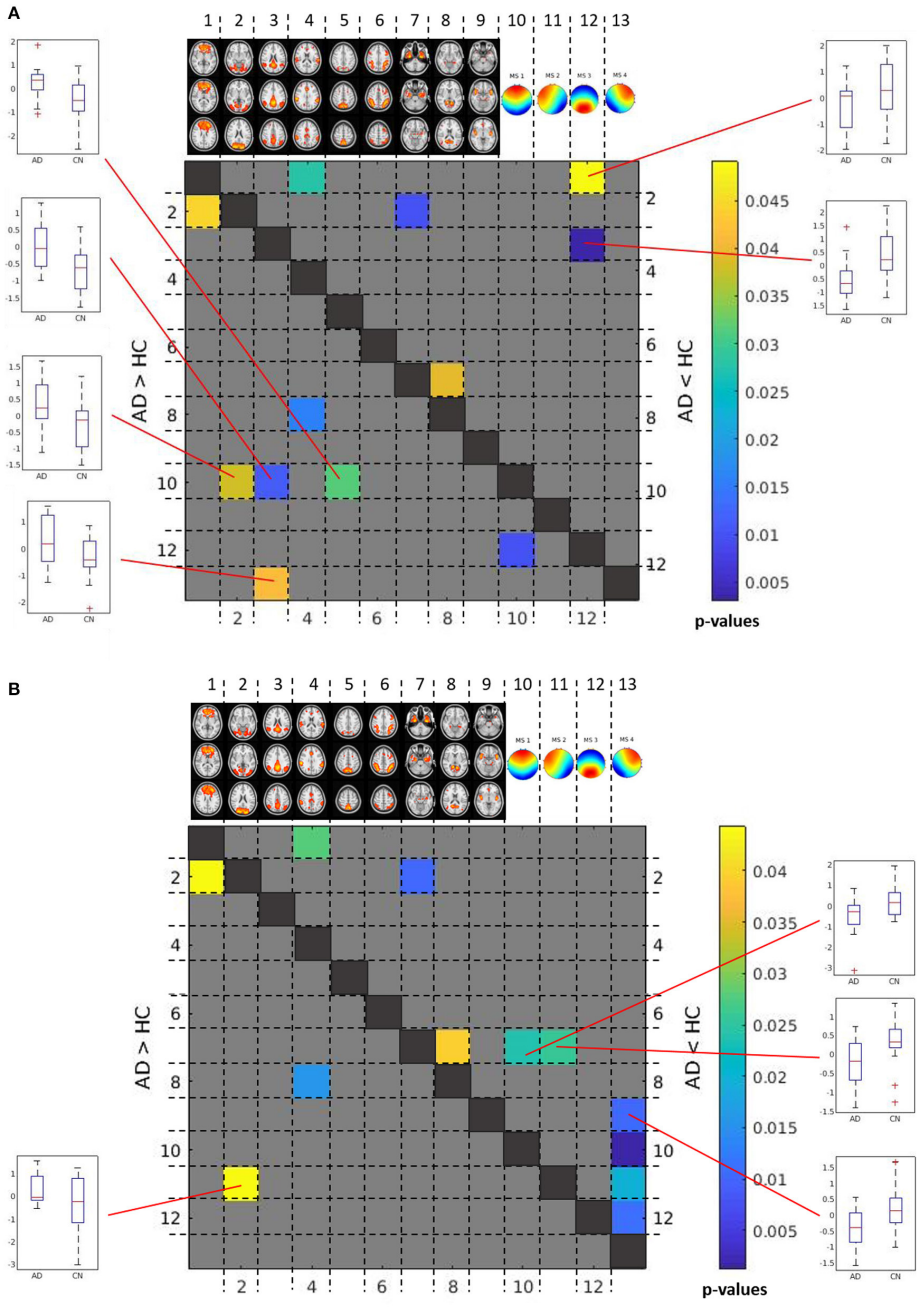
Between group differences in between-network time course correlations. Matrix of differences in correlations between AD patients and controls in Fisher-Z-score correlation coefficients between time courses. The lower left triangular matrix highlights correlations that were less negative or more positive in AD patients than in controls. The upper right triangular matrix highlights correlations that were more positive or less negative in AD patients than in controls. For significant correlation differences between microstate and rs-fMRI component time courses, box plots are shown to illustrate the direction of effects. The color bar indicates uncorrected *p*-values for between group differences. **(A)** Between group differences in between-network correlations based on number of assignments of microstates. **(B)** Between group differences in between-network correlations based on similarity of EEG time courses with each microstate.

Applying an FDR correction, we found no significant differences between AD patients and controls in between-network correlations, neither based on number of classification nor on similarities. Only when using an uncorrected level of significance of *p* < 0.05, we found significant increases and decreases of some correlations as shown in Figure 6.

Based on number of counts, the frontal topology microstate 1 was negatively correlated with the visual network (*p* < 0.04, uncorrected), lateral occipital network (*p* < 0.04, uncorrected), and posterior default mode network (*p* < 0.02, uncorrected), and these negative correlations were stronger in controls than in AD patients. In addition, the negative association of the right anterior topology of microstate 4 with the posterior default mode network was stronger in controls than in AD patients (*p* < 0.05, uncorrected). In contrast, the association of the posterior topology of microstate 3 with the posterior default mode network was positive in controls, but negative in AD patients (*p* < 0.003, uncorrected), while the association of microstate 3 with the frontal lobe network was negative in AD, and almost absent in controls (*p* < 0.05, uncorrected) (Figure 6A).

Based on similarities, the connectivity of the left anterior topology microstate 2 with the visual network was significantly more negative in controls than in AD patients (*p* < 0.05, uncorrected). In contrast, the association of frontal and left lateral anterior topologies microstate 1 and microstate 2 with the bilateral temporal network was significantly more positive in controls than in AD patients (*p* < 0.03 for both comparisons, uncorrected). In addition, the association of the right anterior topology microstate 4 with the anterior salience network was significantly more negative in AD patients than in controls (*p* < 0.02, uncorrected) (Figure 6B).

## Discussion

Here, we studied spatial and temporal associations of EEG microstates with resting state fMRI networks in AD patients and controls. Since 1997, studies have determined microstate alterations in AD dementia and MCI cases, with sample sizes ranging between 20 and 30 cases (21–24, 26). A recent study included almost 200 cases (25). Similar to these previous studies (21, 22, 24, 25) we found an anteriorisation of microstate topologies in our AD patients. In addition, we found strong increases of theta power in AD patients, consistent with previous observations on slowing down of brain rhythms in AD (45). Interestingly, the increase of theta power was homogeneously distributed across the four microstate topologies. This agrees with findings of a previous study (26) and suggests that the microstate changes in the theta band reflect an overall slowing of brain rhythms in AD rather than a topology related effect.

We found that the default mode rs-fMRI network, corresponding to microstate topology 3, was spatially less expressed in AD patients than in controls, particularly in posterior cingulate and temporal lobe regions. This is consistent with findings in a large range of previous rs-fMRI studies [for a systematic review see (46)]. In contrast, spatial expression of the frontal rs-fMRI network, corresponding to microstate topology 1, was increased in mediofrontal and anterior temporal lobe regions in AD compared to controls. The spatial expression of the temporal lobe rs-fMRI network, corresponding to the anterior microstate topologies 2 and 4, was also increased in AD compared with controls. Increases in these networks agree with previous findigns on network abnormalities in AD cases (46, 47). These data suggest that an anteriorisation of the microstate topology in AD was accompanied by corresponding spatial expression changes in resting state fMRI networks. In conclusion, this observation supports the notion that EEG microstates serve as building blocks of brain functional connectivity.

We followed up on this notion using hierarchical clustering to associate time courses of microstate occurrences with their most closely-associated rs-fMRI components. The posterior microstate topology 3 clustered with the default mode rs-fMRI network, and the frontal topology 1 clustered with the frontal lobe rs- fMRI network. The clustering of microstate topology time courses with the time courses of spatially resembling rs-fMRI components support the notion that microstate topologies with a duration of on average 100 milliseconds serve as building blocks of the time courses of resting state networks in rs-fMRI, sampled in the minutes' range. The temporal variation of different topologies allows representing a high number of different brain states despite a limited number of topologies. Importantly, the focus on four microstate topologies represents a simplification as other topologies can be classified as well, even if these account for lower amounts of variance in the EEG time course. Notably, hierarchical clustering is a non-supervised exploratory approach to generate but not to accept hypotheses on associations (48) so that these findings require independent confirmation. In conclusion, combined evaluation of rs-fMRI networks with microstates may allow a more comprehensive characterization of AD related neuronal connectivity changes combining the spatial resolution of fMRI with the temporal resolution of EEG

Following this notion, we determined between-group differences in the strengths of correlations between the time courses of microstate topologies and rs-fMRI networks. We found that the degree of association between the anterior microstate topology 1 and posterior rs-fMRI networks was more negative in controls than in AD patients. This effect did not survive FDR correction, suggesting a potential risk of false-positive findings. If replicated in an independent sample, however, a possible interpretation would be that negative time course correlations are less pronounced in AD cases than in controls. In controls, associations between anterior microstate topologies and posterior rs-MRI components as well as between posterior microstate topologies and anterior rs-MRI components tended to be negative. Negative correlations between posterior and anterior components of rs-fMRI networks have been described before with some caveats due to the role of global signal normalization potentially driving some of these effects (6). Here, we show that such a negative association may also be present between microstate time courses and rs-fMRI components. Previous studies have investigated simultaneous EEG and rs-fMRI acquisitions in a range of conditions, such as sickle cell disease (49), posttraumatic stress disorder (50), narcolepsy (51), and in healthy people (27), but not yet in AD. In addition, these previous studies have not determined associations of time courses between EEG and fMRI data so that our findings expand the current knowledge with regard to the condition (AD) and the temporal associations investigated. Our data point to a predominant loss of negative anterior-posterior correlations of functional brain networks in AD which is reflected not only in rs-fMRI networks, but also in EEG microstates, and supports the notion of a predominant degradation of long reaching intracortical projections in AD.

Our study has several limitations. Firstly, our sample size is relatively small due to high demands on patient preparation and compliance for simultaneous acquisition of EEG and re-fMRI data in people with AD dementia. The presence and size of the effects found in our small sample suggests that these effects should readily replicate in independent samples. Such a replication would add support to the newly-generated hypothesis that time courses of microstate topologies may specifically be associated with time courses of spatially corresponding rs-fMRI components in AD patients and controls. Secondly, the simultaneous EEG-fMRI acquisition led to reduced EEG channels (32 channels), limiting the spatial resolution of our EEG topologies. This simultaneous acquisition of EEG and fMRI data in AD patients and age-matched controls is, however, the crucial, original strength of our study which facilitated direct comparison of the time courses across both modalities. Thirdly, several of the effects did not survive strict multiple comparison correction such as the between-group differences in between-network correlations. But also between group differences in rs-fMRI network expression would not have survived Bonferroni correction for three independent comparisons. The exploratory nature of the analysis and the aim to generate rather than confirm hypotheses on the association between microstates and rs-fMRI pattern justify the reporting and cautious interpretation of findings at only uncorrected levels of significance. Finally, this analysis is not meant to propose that simultaneous EEG-rs-fMRI acquisition will be usefully employed in the diagnostic work up of AD. Rather, the current analysis served to better understand the temporal components underlying rs-fMRI networks and their partial break down in AD.

In summary, we found that time courses of EEG microstates clustered with time courses of spatially corresponding rs-fMRI networks. This supports the notion that the EEG microstates may provide the building blocks of the brain activity that is being sampled by rs-fMRI at a much coarser temporal resolution. The prevalent negative correlations between anterior/posterior microstate topology time courses with posterior/anterior rs-fMRI components replicate earlier findings of anterior-posterior anti-correlations in rs-fMRI data. Of note, in those instances where the AD patients differed from the controls in the degree of association between microstates vs. rs-fMRI networks, these differences was mostly driven by the loss of negative associations in the AD patients. This may represent a possible neurophysiological correlate of the breakdown of long reaching intra-hemispheric connections in AD reported in earlier rs-fMRI studies. Our findings serve to generate the hypotheses that EEG microstates and rs-fMRI activity are correlated and co-occur in AD patients and cognitively healthy age-matched controls. Our data encourage the use of simultaneous EEG and rs-fMRI acquisitions to test the specific spatial associations found in our data.

## Data Availability Statement

The raw data supporting the conclusions of this article will be made available by the authors upon written request, without undue reservation.

## Ethics Statement

The studies involving human participants were reviewed and approved by Ethikkommission der Universitätsmedizin Rostock. The patients/participants provided their written informed consent to participate in this study.

## Author Contributions

ST: designed and conceptualized study, acquisition of the data, analyzed the data, and drafted the manuscript for intellectual content. KB: acquisition of the data and revised the manuscript for intellectual content. AT: helped in analyzing the data, interpreted the data, and revised the manuscript for intellectual content. KJ: helped in analyzing the data and revised the manuscript for intellectual content. M-AW: acquisition of the data and revised the manuscript for intellectual content. CB: analyzed the data and revised the manuscript for intellectual content. All authors contributed to the article and approved the submitted version.

## Conflict of Interest

ST participated in scientific advisory boards of Roche Pharma AG, Biogen, and MSD, and received lecture fees from Roche and MSD. KB currently is an employee of Springer Medizin Verlag GmbH, Munich, Germany (subsidiary of Springer Nature, Berlin, Germany). The work reported here was conducted before she took her position at Springer, when she still was affiliated with the DZNE. The remaining authors declare that the research was conducted in the absence of any commercial or financial relationships that could be construed as a potential conflict of interest.
