# Supplementary material for: Simultaneous Assessment of Electroencephalography Microstates and Resting State Intrinsic Networks in Alzheimer's Disease and Healthy Aging
Source: Front Neurol. 2021 Jun 17;12:637542. doi: 10.3389/fneur.2021.637542 (PMC8249002; doi:10.3389/fneur.2021.637542)

Supplementary Figure 1: Hierarchical classification of rs-fMRI components' and microstates' time courses based on their cross-correlation across individuals in controls only

Cluster dendrograms for the time courses of rs-fMRI components and the microstates. The lower left triangular matrix indicates the cross-correlation of time courses, the upper right triangular matrix the partial correlations of time courses.

Clustering was performed only across the controls.

Supplementary Figure 1a: Clustering based on number of assignments of microstates per TR

Supplementary Figure 1b: Clustering based on similarity of EEG time courses with each microstate per TR

Supplementary Figure 1: Hierarchical classification of rs-fMRI components' and microstates' time courses based on their cross-correlation across individuals in controls only

Supplementary Figure 1a: Clustering based on number of assignments of microstates per TR

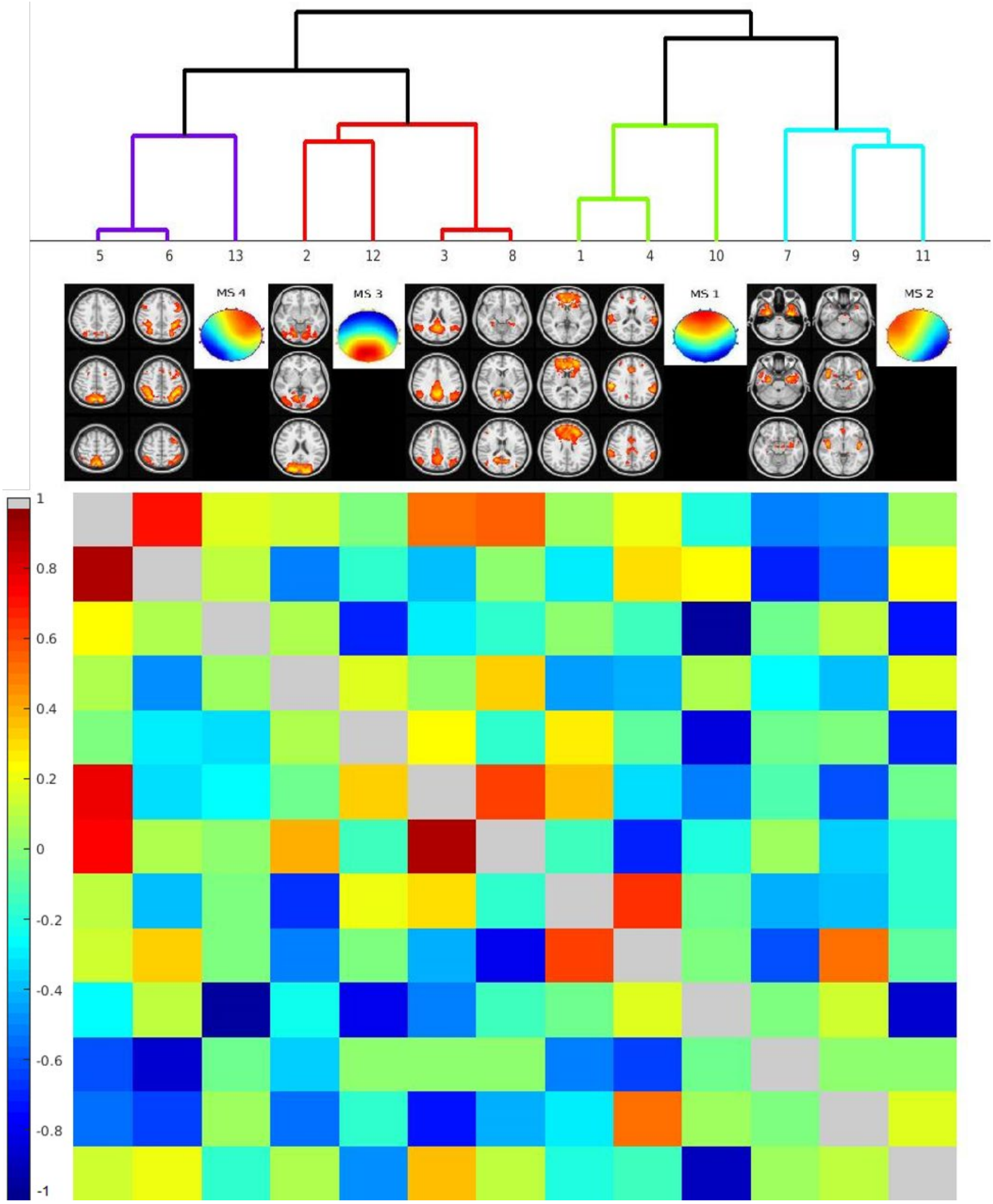

Supplementary Figure 1b: Clustering based on similarity of EEG time courses with each microstate per TR

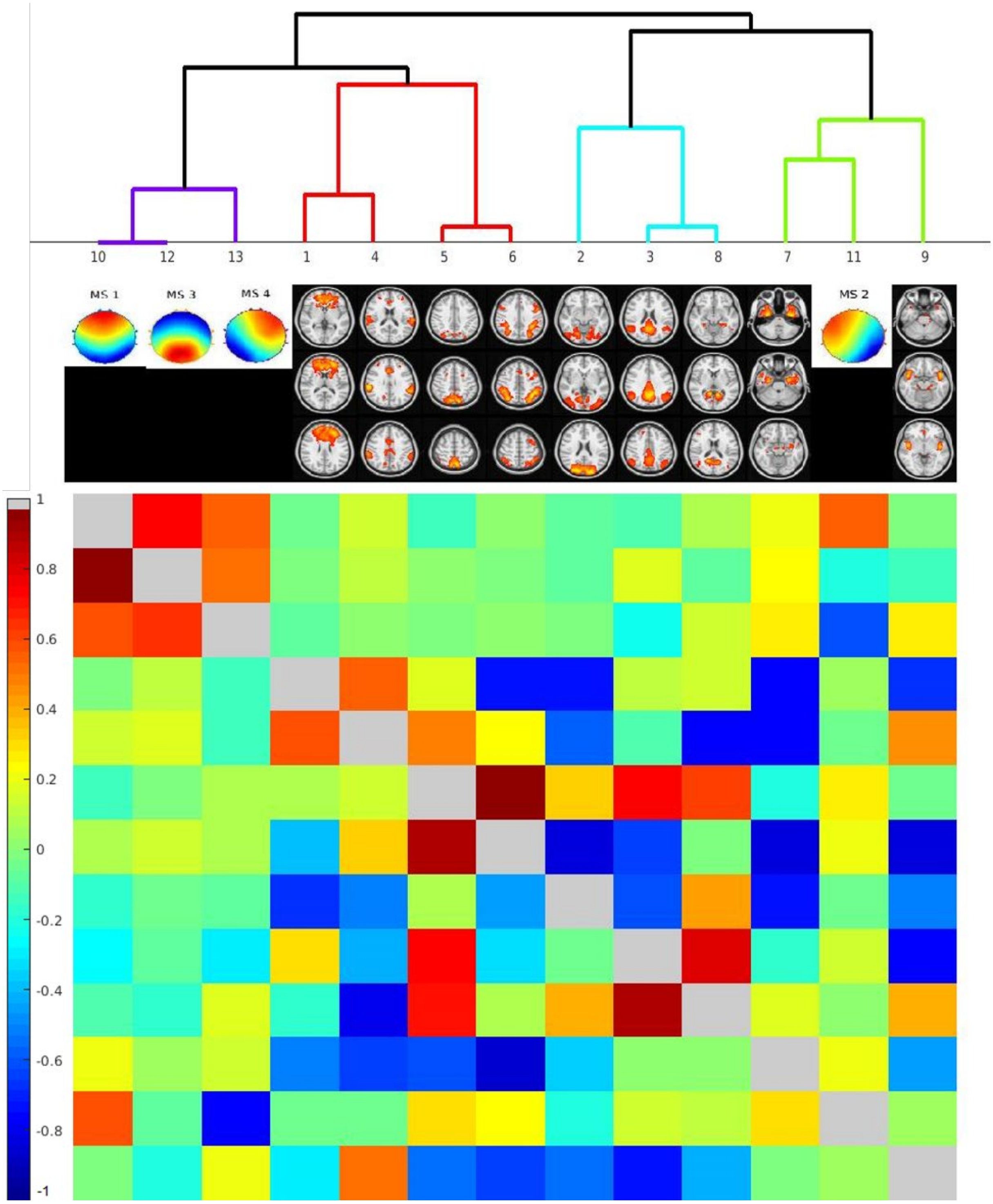

Supplement: Supplementary file 1 [file Image_1.pdf]
